# Percutaneous tracheostomy: Comparison of three different methods with respect to tracheal cartilage injury in cadavers—Randomized controlled study

**DOI:** 10.3389/pore.2023.1610934

**Published:** 2023-01-19

**Authors:** Fruzsina Bódis, Gábor Orosz, József T. Tóth, Marcell Szabó, László Gergely Élő, János Gál, Gábor Élő

**Affiliations:** ^1^ Department of Otorhinolaryngology and Head and Neck Surgery, Semmelweis University, Budapest, Hungary; ^2^ Department of Anaesthesiology and Intensive Therapy, Semmelweis University, Budapest, Hungary; ^3^ Department of Surgery, Transplantation and Gastroenterology, Semmelweis University, Budapest, Hungary

**Keywords:** percutaneous tracheostomy, tracheal cartilage injury, Tracheal stenosis, Griggs, Ciaglia Blue Rhino

## Abstract

**Background:** Performing tracheostomy improves patient comfort and success rate of weaning from prolonged invasive mechanical ventilation. Data suggest that patients have more benefit of percutaneous technique than the surgical procedure, however, there is no consensus on the percutaneous method of choice regarding severe complications such as late tracheal stenosis. Aim of this study was comparing incidences of cartilage injury caused by different percutaneous dilatation techniques (PDT), including Single Dilator, Griggs’ and modified (bidirectional) Griggs’ method.

**Materials and methods:** Randomized observational study was conducted on 150 cadavers underwent post-mortem percutaneous tracheostomy. Data of cadavers including age, gender and time elapsed from death until the intervention (more or less than 72 h) were collected and recorded. Primary and secondary outcomes were: rate of cartilage injury and cannula malposition respectively.

**Results:** Statistical analysis revealed that method of intervention was significantly associated with occurrence of cartilage injury, as comparing either standard Griggs’ with Single Dilator (*p* = 0.002; OR: 4.903; 95% CI: 1.834–13.105) or modified Griggs’ with Single Dilator (*p* < 0.001; OR: 6.559; 95% CI: 2.472–17.404), however, no statistical difference was observed between standard and modified Griggs’ techniques (*p* = 0.583; OR: 0.748; 95% CI: 0.347–1.610). We found no statistical difference in the occurrence of cartilage injury between the early- and late post-mortem group (*p* = 0.630). Neither gender (*p* = 0.913), nor age (*p* = 0.529) influenced the rate of cartilage fracture. There was no statistical difference between the applied PDT techniques regarding the cannula misplacement/malposition.

**Conclusion:** In this cadaver study both standard and modified Griggs’ forceps dilatational methods were safer than Single dilator in respect of cartilage injury.

## Introduction

Prolonged invasive mechanical ventilation of a patient *via* orotracheal tube may cause several early and late intubation-associated complications including pneumonia, pressure ulcers of the surrounding tissues, tracheal stenosis, etc. It is also highly responsible for patient discomfort due to swallowing incompetence, impaired communication ability and poor mouth hygiene. To avoid these complications and enhance patient comfort currently it is widely accepted to perform either surgical or percutaneous tracheostomy for patients who need prolonged mechanical ventilation (1–4).

The first widely accepted percutaneous tracheostomy technique was described by Pat Ciaglia, which involved a series of sequential dilatations using a set of seven dilators of progressively larger size (5). A variant of this technique, using a single tapered dilator is called the Ciaglia Blue Rhino^TM^ (Cook Medical, Bloomington IN, United States) method which is now most commonly used, especially in the United States and United Kingdom (6). There is a very similar method to this when a balloon is introduced over a guidewire to dilate the stoma (7). In PercuTwist^TM^ (Teleflex, Athlone, Ireland) technique, a specially designed screw-type dilator is introduced over a guidewire and its rotation dilates the stoma (8). Another widely used technique was developed by William Griggs who suggested to use specifically modified forceps enabling the performance of the main dilation in a single step (9). The other great advance of this technique is that all the aforementioned methods require pressure over the anterior wall during dilation risking a possible posterior tracheal wall injury (6). An interesting alternative was described by Fantoni, where the dilator and the tracheostomy tube are pulled through the larynx in a retrograde fashion (10).

The spread of percutaneous techniques raised the number of successful bedside tracheostomy operations, and percutaneous dilatational tracheostomy (PDT) gained popularity to become the technique of choice for long-term airway management in mechanically ventilated patients. However, these interventions have their own hazards: the major early complication is hemorrhage and one of the most dangerous and relatively common late complication is tracheal stenosis. Severe tracheal stenosis occurs in 3%–12% of patients undergoing PDT (11, 12) which is considered an excruciating late complication of tracheostomy with significant impact on quality of life. Mechanisms of postintubational and posttracheostomy tracheal stenosis are not clearly identified. After a long term intratracheal intubation the cause could be ischaemia of the mucous membrane, pressure ulceration or granulation tissue, while in case of trauma tracheal stenosis is rather caused by cartilage injury as it was described in trauma patients and in canine model (13–19). A. Marchion et al published a molecular, histolgical study in 2022. They found that the reason could be aberrant wound-healing, fibrotic scarring and iatrogenic aetiology as trauma of the tracheal mucosa, and of the tracheal cartilage; (20). As there is a possible causative relationship between the fracture of the cartilage during the PDT procedure and the aforementioned tracheal stenosis, in this study we wanted to investigate how common cartilage fracture is when performing PDT on cadavers with the two most common methods, namely Griggs’ and single dilator (Rhino), and we also reevaluated and compared our previously described modified Griggs’ method (in which we make a wider incision, blunt preparation, and bidirectional forceps dilation of the tracheal wall, e.g. standard horizontal and novel vertical) with them (21). The further late complications such as tracheomalacia, tracheoinominate artery fistula and tracheoesophageal fistula occur each in <1%. Tracheaomalacia usually caused by the high cuff pressure of the tracheal tube. This and the intraoperational perforation of the posterior tracheal wall could also be the cause of the tracheoesophageal fistula. If the tracheostomy tube is positioned under the 4th tracheal cartilage the risk of the development of the tracheoinominate artery fistula is higher. These late complications cannot be investigated on cadavers (12–27).

## Materials and methods

Our cadaver study was performed at the 1st Department of Pathology and Experimental Cancer Research of Semmelweis University with permission of Semmelweis University Regional and Institutional Committee of Science and Research Ethics (117-1/2006 SE-RKEB; 2014.03.17). Data were collected anonymized. Data analysis and statistical plan was written and filed with a private entity before data were accessed.

The simple-blinded observational method was carried out by two experts in the field of surgical and percutaneous tracheostomies—by an intensive care specialist and an ENT surgeon with additional specialization of intensive care medicine—both for more than 5 years at a University hospital. The first expert had performed the procedures and the second one had checked the results, respectively.

Cadavers (who were not intubated before death) were collected; autopsies as well as preparation of the airway complexes from the cadavers following tracheostomy were carried out by mortuary technician. Site of tracheal cannula insertion, estimation of tracheal cartilage damage were scored by the non-operator expert without the knowledge of the insertion technique.

Insertion site was scored as 1: cricoid-1st tracheal ring space; 2: 1st-2nd tracheal ring space; 3: 2nd-3rd tracheal ring space. Estimation of ring injuries was classified as: 1: none; 2: one ring fractured; 3: two or more rings fractured.

Data about the cadavers [e.g., date and time of death (72 h or more before intervention), age, gender] as well as the lack of exclusion criteria (e.g., previous laryngo-tracheal disease, injury, previous tracheostomy, visible or palpable damage on the neck) were collected and recorded on individual sheets with registration numbers.

Randomization was carried out by a 6-sided dice: Nr 1-2, 3-4 and 5-6 were linked with traditional Griggs (*n* = 51), modified Griggs (*n* = 55) and Single Dilator methods (*n* = 44), respectively.

Cadavers were positioned in standard hyperextended position for tracheostomy, and the exact method was performed according to the randomization. Details of the procedures are briefly presented below [13] and were discussed previously elsewhere. [14] Percutaneous tracheostomy kits available from the market were used. For traditional and modified Griggs’ technique Portex® GRIGGS© Percutaneous Dilation Tracheostomy Kit with Blue Line Ultra® tubes in different sizes were used according to the needs. For Ciaglia method Portex® ULTRAperc© Single Stage Dilator Technique Kit with Blue Line Ultra® tubes were applied in the same manner.

Skin incisions were performed at the midpoint of cricoid cartilage and jugulum. Traditional Griggs’ technique:, no surgical preparation, direct tracheal puncture and unidirectional (transverse) tracheal dilation were performed followed by the insertion of tracheostomy cannula (9).

Modified Griggs’ technique: a maximum 50 mm-wide skin incision, surgical pretracheal tissue preparation, and careful, visualized bidirectional (transverse and longitudinal) intercartilaginous forceps dilation of tracheal wall were performed (21). The wider horizontal skin incision provided enough space for secure pretracheal preparation until the tracheal wall could be visualized as well as palpated in our modified technique. Preparations were performed using blunt technique, and Griggs-modified dilating forceps were used in order to ascertain tissue resistance followed by the tracheal puncture according to Watters recommendations (28). Visualized tracheal wall puncture next to the palpating finger and continuous aspiration of air secured correct intratracheal needle position according to Paran’s recommendation (29). Insertion of the guidewire followed by the introducer was the following step. Griggs-modified dilating forceps were introduced using the guidewire, according to the standard method, as deep as approximately 15 mm from the axis of rotation, and bimanual horizontal dilation was performed. Forceps were rotated 90° in longitudinal direction afterwards, and another bimanual forceps dilation was carried out in the vertical direction. After removing the forceps tracheostomy tube was inserted into the trachea using the guidewire.

The Ciaglia Blue Rhino single dilator method was performed as reviewed by SP Ambesh (30). After a transverse 20 mm skin incision we identified the anterior tracheal wall by palpation to assess the puncture site. The tracheal dilation was performed by a curved, gradually tapered dilator in a single step, than we inserted the tracheostomy tube *via* using a guidewire.

Upper airway complex was anatomized by a mortuary technician with maximal care for non-traumatic extraction. Site of insertion and posterior tracheal wall were inspected and recorded by the non-operator expert, then dissection of the posterior wall was carried out. Exact cannula position was checked and ring fracture inspected and palpated to avoid any “hidden” fracture covered by mucosa. The process was completed by photo documentation.

### Statistics

Pearson chi-square tests were applied using SigmaStat 3.5 program (Systat Software Inc., San Jose, CA, United states) for statistical evaluation. To calculate the sample size of the study, the frequency of tracheal cartilage injury was the variable of interest. We assumed that a minimum of 20% difference was considered clinically important, and that in combination with our previous results of 9% from earlier experiments were used for the calculations. [13] A type one error of 0.05 and a required (statistical) power of 0.80 were set. A minimum of 150 cadavers were required based on the conditions detailed above.

## Results

We carried out PDTs in 150 cadavers (69 female and 81 male) with different types of dilatational techniques in the period of 2015 March 01–2017 February 24. All data is available in Supplementary Material. Standard Griggs technique was applied in 51 cases, modified Griggs technique in 55 cases and Single Dilator technique in 44 cases according to randomization. During autopsy, we found that in four cases the tracheostomy tube was misplaced - not in the trachea but in the surrounding tissues (all done by Single Dilator method). We excluded these results; thus statistical analysis consists of 146 cases.

**FIGURE 1:**
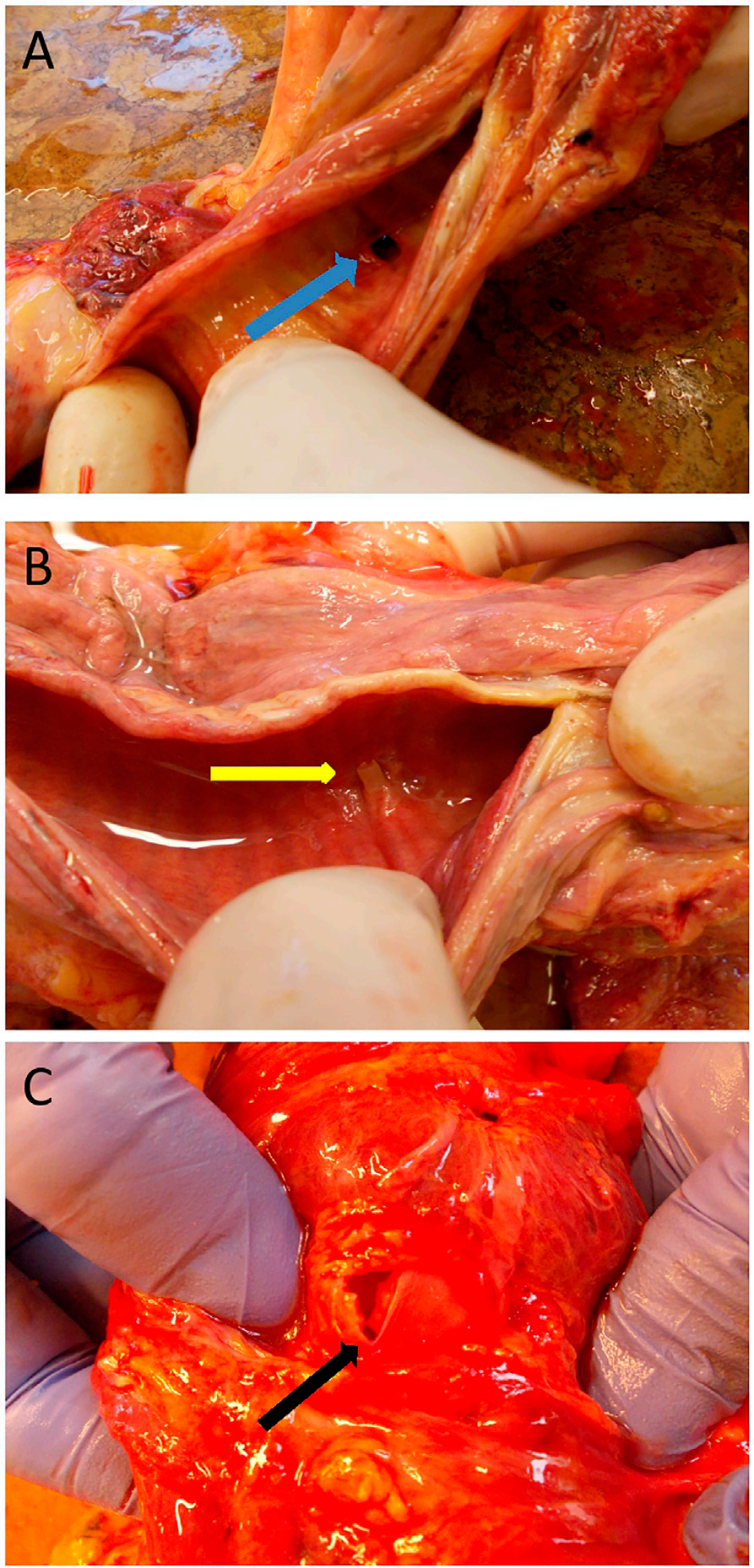
Characteristic of tracheal injuries during autopsies **(A)**: Tracheostomy aperture [blue arrow] without fracture (posterior tracheal wall dissected, internal view). **(B)** “One-ring” fracture [yellow arrow] (posterior tracheal wall dissected, internal view). **(C)** “Multiple-ring” fracture [black arrow] (anterior view for demonstrational purposes).

According to the time elapsed since death we divided the patients into two groups, an early post-mortem (less than 72 h passed since death), and a late post-mortem group (81 vs. 65 subjects). Our preliminary assumption was that cartilage rigidity varies with the time elapsed after death thus there would be an increase in cartilage injury in the group of cadavers in whom PDT was carried out more than 72 h after death (late post-mortem group). Table 1 shows that the frequency of cartilage injury was 53.09% (43/81 cases) and 58.46% (38/65 cases), respectively, hence there was no statistical difference between these two groups (*p* = 0.630). Figure 1 presents characteristic images of tracheal injuries found during autopsies. Thus for further analysis we summarized all subjects irrespective of the time elapsed from death until PDT was carried out. We also recorded the age of subjects at death and according to this we compared cartilage injury occurrence in subjects younger (34/71) and older than 70 years (31/75). No statistical difference was found between these two groups (*p* = 0.529), thus cartilage fracture does not depend on the age of the deceased. There was also no statistical difference (*p* = 0.913) in the cartilage injury occurrence between women (29/67) and men (36/79).

**TABLE 1:** Relationship between cartilage injury and study variables.

	No injury	Injury	Injury (%)	*p*-value
early post-mortem (less than 72h after death)	38	43	53.09	0.630
late post-mortem (more than 72h after death)	27	38	58.46	
cadaver younger than 70 years	34	37	52.11	0.529
cadaver older than 70 years	31	44	58.67	
women	29	38	56.72	0.913
Men	36	43	54.43	

As the next step we compared the three techniques applied according to two aspects, whether the occurrence of cartilage injury depends on the dilatational techniques and whether there is any difference in the position of tracheostomy. For the latter we accepted the tracheostomy at “proper position” if the tube was inserted between the 1st and 2nd or between the 2nd and 3rd cartilages according to the standards. Raw data are shown in Table 2. When performing tracheostomy with standard Griggs’ technique, there was no cartilage injury observed in 26 cases, and injured cartilages were detected in 25 cases (injury occurrence: 49.02%), meanwhile 31 tubes were inserted in good position and 20 were malpositioned (malposition occurrence: 39.22%). In case of modified Griggs’ method these results are 32 vs. 23 (41.82%) for injury and 36 vs. 19 (34.55%) for malposition, and 7 vs. 33 (82.50%) and 22 vs. 18 (45.00%), respectively, when using Single Dilator.

**TABLE 2:** Relationship between cartilage injury or tracheostomy tube position and the method of intervention.

Method	No injury	Injury	Injury (%)	Good position	Bad position	Malposition (%)
Griggs’	26	25	49.02	31	20	39.22
modified Griggs’	32	23	41.82	36	19	34.55
Single Dilator	7	33	82.50	22	18	45.00

Statistical analysis revealed that the method of intervention was significantly associated with cartilage injury, when comparing either standard Griggs’ with Single Dilator (*p* = 0.002; OR: 4.903; 95% CI: 1.834–13.105) or modified Griggs’ with Single Dilator (*p* < 0.001; OR: 6.559; 95% CI: 2.472–17.404) technique. In each case we found that there is an increased risk of cartilage injury when using the Single Dilator technique. However, we could not detect any statistical difference between standard and modified Griggs’ techniques with respect to cartilage injury (*p* = 0.583; OR: 0.748; 95% CI: 0.347–1.610). We also analyzed the malposition occurrence, but there was no statistically significant difference between the three PDT techniques applied (Griggs vs. modified Griggs: *p* = 0.767; OR: 0.818; 95% CI: 0.371–1.803; Griggs vs. Single Dilator: *p* = 0.733; OR: 1.268; 95% CI: 0.548–2.935; and modified Griggs vs. Single Dilator: *p* = 0.413; OR: 1.550; 95% CI: 0.673–3.572). Statistical data are shown in Table 3.

**TABLE 3:** Statistical analysis of the methods of intervention according to cartilage injury and tracheostomy tube position.

	Cartilage injury			Malposition		
	*p*-value	odds ratio	CI 95%	*p*-value	odds ratio	CI 95%
Griggs’ vs. modified Griggs’	0.583	0.748	0.347 to 1.610	0.767	0.818	0.371 to 1.803
Griggs’ vs. Single Dilator	0.002	4.903	1.834 to 13.105	0.733	1.268	0.548 to 2.935
modified Griggs’ vs. Single Dilator	<0.001	6.559	2.472 to 17.404	0.413	1.550	0.673 to 3.572

## Discussion

Tracheostomy was traditionally performed by surgeons in the operating room, but thanks to the development of percutaneous dilatational techniques nowadays is a routine procedure done by intensivists in most of the Intensive Care Units. PDT helps shorten overall time of tracheostomy procedures by decreasing the waiting time for operating room and general anesthesia (31–33). Another great review also suggests that PDT is the procedure of choice for tracheostomy because of its reduced procedure time and medical cost (34) There are several good quality prospective randomized studies which confirm the PDT’s superiority against surgical techniques in critically ill patients (35–38). PDT is associated with significantly fewer wound infections and unfavourable scarring, and it may also reduce the risk of bleeding and mortality compared with surgical tracheostomy in critically ill patients according to systematic reviews (39, 40). Bedside percutaneous tracheostomy was experienced as a safe method in a retrospective review of more than 3000 procedures (41). However, in a recent study, there was no statistically significant difference in the one-year mortality of patients undergoing prolonged mechanical ventilation whether receiving tracheostomy or not (42), furthermore there were no significant differences in the intraoperative and postoperative bleeding and in mortality in recent meta-analysis (43).

Although there are several studies, meta-analyses and excellent reviews which compare the different PDT techniques (6,30,44–47), there is still no consensus between physicians on which technique has the lowest major complication rate. Our aim in this study was to compare the two most widely used PDT techniques—namely the Griggs’ and the Single Dilator—and also our previously suggested modification of Griggs’ version (21) in cadavers, in respect of cartilage injury, which is believed to take important part in the development of tracheal stenosis (48, 49).

According to our data, using a single tapered dilator significantly increases the risk of cartilage injury in cadavers compared to both standard and modified Griggs’ methods, in accordance with some previous experts’ observations (30). The single dilator is a round shaped device with permanently increasing diameter which is used with a continous force against the tracheal wall therefore the trachea cartilage could be broken easier in this case. A mild single unidirectional (transverse) tracheal dilation is performed in Griggs method, while performing modified Griggs technique a visualized bidirectional (transverse and longitudinal) intercartilaginous dilation is made which both can reduce the risk of cartilage injuries (50).

We have suggested a modification to the classical Griggs’ method in order to decrease the complications of PDT in our previous study (21). We have found that this modification caused a remarkable decrease in the number of injured tracheal cartilages. One of the main drawbacks of that study was that the procedures were not blinded, i.e., the same investigator performed and evaluated identical procedures. Thus, now we redesigned and reevaluated our previous study using a randomized, blinded approach. Our new data still showed a decreased risk of cartilage injury with this modification; however, this reduction was not statistically significant. Further elevation of the sample size may emphasize the difference. Cartilage fracture was independent of aging, gender and the time elapsed since death. According to statistical analysis the level of intervention was not different between the three groups, however, it is worth mentioning that cannula malposition occurred less commonly in the modified Griggs’ group.

We suggest making a quite wide (50-mm) horizontal skin incision for the procedure. One might say that this increases the risk of major bleeding and makes the procedure longer but at the same time it provides enough space for secure pretracheal surgical blunt preparation until the tracheal wall can be visualized and palpated making critical intervention (i.e. tracheal puncture with partially lost airway) shorter and probably safer. Another important aspect is, that with good visibility bronchoscopy is nearly unnecessary, thus it is easier to avoid the significant rise of PaCO_2_ and consequent respiratory acidosis which occurs during bronchoscopy even when applying increased tidal volume and large diameter endotracheal tube (51).

The obvious limitation of our study is that PDTs were made in cadavers, which could influence cartilage flexibility, so cadaver’s tracheal cartilages could be more rigid than in case of living tissue. Performing PDT cartilage injury/fracture could be rare in living tissues but the correlation of the number of the cartilage injuries using different methods could be demonstrated on cadavers.Tracheal injuries were more common in our study (81/146 subjects, 55.48%) than what we would expect according to our clinical experience, although it was still less than mentioned in another PDT cadaver study (83%) (52). Even if there was no significant difference in cartilage injury between the two groups defined by the post-mortem time elapsed, there was a higher incidence in the late post-mortem group, which corroborates the presumption that it could influence our data. It is also important to mention that cartilage injury could not only occur during the insertion of the cannula but also during autopsy when it was removed for evaluation, thus it could further increase its incidence.

In conclusion, in cadavers we found that forceps dilatational methods are safer than Single dilator with respect to cartilage injury. Although we did not detect statistical significance, we still suggest the use of bidirectional dilatation with forceps, as cartilage injury and cannula malposition incidence was the lowest in the group where this modification was applied. Prospective study and long-term follow-up of patients undergoing PDTs would be necessary to further confirm its superiority over other methods.

## Data Availability

The original contributions presented in the study are included in the article/Supplementary Material, further inquiries can be directed to the corresponding author.

## Supplementary material

SUPPLEMENTARY DATA SHEET S1 The generated datasets.
